# Clonal expansion of mtDNA deletions: different disease models assessed by digital droplet PCR in single muscle cells

**DOI:** 10.1038/s41598-018-30143-z

**Published:** 2018-08-03

**Authors:** Selena Trifunov, Angela Pyle, Maria Lucia Valentino, Rocco Liguori, Patrick Yu-Wai-Man, Florence Burté, Jennifer Duff, Stephanie Kleinle, Isabel Diebold, Michela Rugolo, Rita Horvath, Valerio Carelli

**Affiliations:** 1 Unit of Neurology Department of Biomedical and Neuromotor Sciences (DIBINEM), University of Bologna, Bologna, Italy; 2 Wellcome Trust Centre for Mitochondrial Research, Institute of Genetic Medicine, Newcastle University, Newcastle upon Tyne, NE1 3BZ UK; 3 IRCCS Institute of Neurological Sciences of Bologna (ISNB), Bellaria Hospital, Bologna, Italy; 4 NIHR Biomedical Research Centre at Moorfields Eye Hospital and UCL Institute of Ophthalmology, London, UK; 5 Cambridge Centre for Brain Repair, Department of Clinical Neurosciences, and MRC Mitochondrial Biology Unit, University of Cambridge, Cambridge, UK; 6 Medical Genetic Center Munich, 80335 München, Germany; 7 Department of Pharmacy and Biotechnology, University of Bologna, Bologna, Italy

## Abstract

Deletions in mitochondrial DNA (mtDNA) are an important cause of human disease and their accumulation has been implicated in the ageing process. As mtDNA is a high copy number genome, the coexistence of deleted and wild-type mtDNA molecules within a single cell defines heteroplasmy. When deleted mtDNA molecules, driven by intracellular clonal expansion, reach a sufficiently high level, a biochemical defect emerges, contributing to the appearance and progression of clinical pathology. Consequently, it is relevant to determine the heteroplasmy levels within individual cells to understand the mechanism of clonal expansion. Heteroplasmy is reflected in a mosaic distribution of cytochrome *c* oxidase (COX)-deficient muscle fibers. We applied droplet digital PCR (ddPCR) to single muscle fibers collected by laser-capture microdissection (LCM) from muscle biopsies of patients with different paradigms of mitochondrial disease, characterized by the accumulation of single or multiple mtDNA deletions. By combining these two sensitive approaches, ddPCR and LCM, we document different models of clonal expansion in patients with single and multiple mtDNA deletions, implicating different mechanisms and time points for the development of COX deficiency in these molecularly distinct mitochondrial cytopathies.

## Introduction

Mitochondria are cytoplasmic organelles crucially involved in respiration and energy production of eukaryotic cells, unique for containing their own maternally inherited genome^1^. Human mitochondrial DNA (mtDNA) is a small, circular, intronless 16.6 kb long molecule, encoding 37 genes, precisely 13 proteins of the oxidative phosphorylation (OXPHOS) system, 22 transfer RNAs (tRNAs) and 2 ribosomal RNAs (rRNAs). Furthermore, mtDNA is a high copy number genome and mitochondrial mass varies amongst different cell and tissue types depending on the metabolic demands^2^.

The first mtDNA mutations associated with human diseases were deletions in patients with mitochondrial myopathies^3^ and a missense point mutation in patients with Leber’s hereditary optic neuropathy^4^. The mtDNA deletions remove various portions of the genome, resulting in shorter molecules, and they may occur either as single or multiple deletions^5,6^.

Single, large-scale deletions cause sporadic diseases, which may start in early childhood such as Pearson and Kearns-Sayre syndromes or, later in life, such as chronic progressive external ophthalmoplegia (CPEO)^7^. Single mtDNA deletions are considered sporadic, somatic mutational events occurring early during embryonic development in the majority of cases. However, the 4977bp “common deletion” was found to exist at very low heteroplasmic loads in unfertilized oocytes^8–10^. Interestingly, a few cases of maternally inherited single mtDNA deletions have been reported and considered rare exceptions^10–14^.

Differently, multiple mtDNA deletions may accumulate somatically in post-mitotic tissues secondary to mutations in nuclear genes inherited as Mendelian traits, directly involved in mtDNA replication, or in the nucleotide pool balance or, more recently, in mitochondrial dynamics^15^. Thus, mtDNA deletions generated by different genetic defects may propagate within muscle fibers with different modes and timing.

Deleted mtDNA molecules coexist with wild-type mtDNA, resulting in a heterogeneous intracellular pool of mitochondrial genomes known as heteroplasmy^2^. Over time, some of the deleted mtDNA species can accumulate to high loads due to clonal expansion, which may occur both in germline and somatic cells. Thus, heteroplasmy may vary amongst cells and tissues, impinging on the phenotypic expression of mitochondrial disorders, and clonal expansion becomes the driving force, as deleted mtDNA molecules become the prevalent population within single cells^16^. The mechanisms beyond this process have not been fully determined due to technical limitations. Crucially, the investigation of clonal expansion patterns requires a precise quantitative assay to determine the proportion of deleted and wild type mtDNA molecules at the single cell level. This assay should be performed at different locations of the same cell, according to histochemical demonstration of mitochondrial dysfunction, such as muscle fiber domains deficient for cytochrome *c* oxidase (COX). Recently, a sensitive and powerful technique, droplet digital PCR (ddPCR), has been developed for quantifying deleted mtDNA and it was demonstrated to be more precise compared with conventional real-time quantitative PCR (qPCR) techniques. A major benefit of ddPCR in heteroplasmy quantification is that it does not require external standards, thus limiting the error rates. Partitioning of samples, as in single molecule PCR, brings enhanced sensitivity and the final reaction is visualized using fluorescence rather than gel analysis^17,18^.

To better understand the chronology and mechanisms driving clonal expansion of deleted mtDNA molecules, we used the ddPCR approach to analyze single muscle cells collected by laser-capture microdissection of both transversal and longitudinal sections. We compared three different molecular paradigms of disease, i.e. single large-scale mtDNA deletions and multiple DNA deletions caused by nuclear mutations in the *OPA1* (Optic Atrophy 1) or *POLG* (Polymerase **γ**) genes.

## Materials and Methods

### Patients

We studied 7 patients presenting with three different pathologies associated with accumulation of mtDNA deletions in the skeletal muscle (Table 1). Patients 1 and 2 harbored a dominant mutation in the *OPA1* gene and were clinically affected by a dominant optic atrophy (DOA) “plus” syndrome; patients 3 and 4 carried recessive mutations in the *POLG* gene and were clinically affected by SANDO syndrome (Sensory Ataxic Neuropathy, Dysarthria, Ophthalmoparesis); finally, patients 5, 6 and 7 were sporadic cases carrying a single mtDNA deletion (two with the “common deletion” and one with a 5992bp deletion) and affected with CPEO. We aimed to represent three different categories of mitochondrial disorders: patients with *OPA1* mutations may accumulate mtDNA multiple deletions because of defective mitochondrial dynamics, patients with *POLG* mutations present with pathological mtDNA maintenance due to defective mtDNA replication, and patients with single mtDNA deletion are the paradigm of sporadic events of somatic deletion. We also studied 3 age-matched individuals with normal muscle histology and histochemistry as controls (Table 1). A brief description of the patients is provided in the Supplemental Material. Patients 1 and 2 were previously reported^19,20^. Additionally, we have investigated blood DNA samples from 16 patients with Pearson and Kearns Sayre syndromes or CPEO, for the presence of mtDNA deletions, and a brief description of these patients is reported in Table S1. For all cases informed consent and a muscle biopsy was obtained, and the local Ethics Committee “Comitato Etico Interaziendale Bologna-Imola-Ferrara (CE-BIF) #13036” approved the study. All experiments and methods were performed in accordance with relevant guidelines and regulations. Each case has been molecularly defined by standard approaches such as Southern blot or quantitative real time PCR, and the heteroplasmic load of single/multiple deletions is reported in Table 1.

**Table 1:** Characteristics of muscle biopsy samples used in this study.

Case and phenotype	Genetic defect	Age at time of biopsy	Sex	Nuclear gene mutation or mtDNA deletion breakpoints	AA change	Heteroplasmy estimation of muscle biopsy DNA
P1 (DOA plus)	*OPA1* heterozygous missense	66	M	*OPA1* *c.1462G>A*	p.G488R	56% (Southern blot)
P2(DOA plus)	*OPA1* heterozygous missense	42	M	*OPA1* *c.1316G>T*	p.G439V	37% (Southern blot)
P3(SANDO)	*POLG* heterozygous missense and insertion	53	M	*POLG1* *c.934T>C* *c.3629insA*	p.W312R and p.Y1210X	76% (Southern blot)
P4 (SANDO, Parkinsonism)	*POLG* homozygous missense	57	F	*POLG1* *c.1943C>G*	p.P648R	64% (Southern blot)
P5(CPEO)	mtDNA single deletion^a^	49	M	mtDNA nt.8469-nt.13447	NA	NA
P6 (CPEO)	mtDNA single deletion^a^	18	F	mtDNA nt.8469-nt.13447	NA	74% (Southern blot)
P7 (CPEO)	mtDNA single deletion	50	M	mtDNA nt.9431-nt.15423	NA	NA
C1	Control 1	37	F	NA	NA	NA
C2	Control 2	20	F	NA	NA	NA
C3	Control 3	41	F	NA	NA	NA

AA, amino acid; F, female; M, male; NA, not applicable.

^a^Common deletion.

### Transversal and longitudinal muscle biopsy sectioning

**Figure 1:**
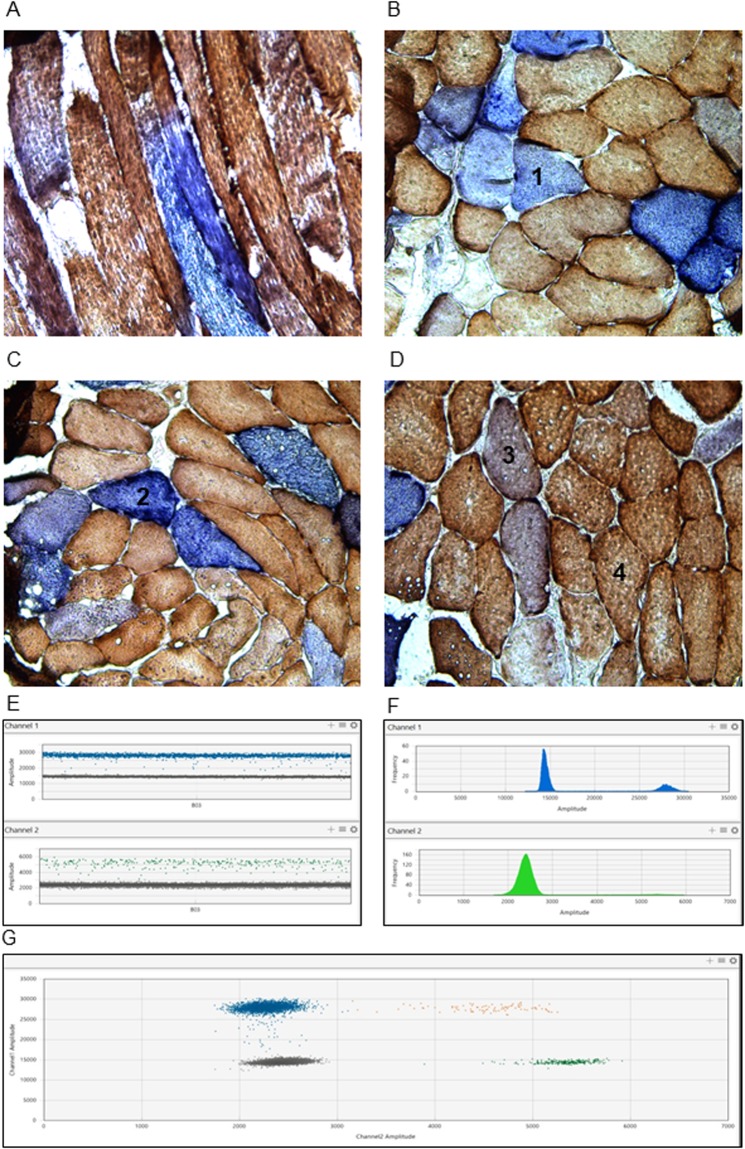
Representative muscle biopsies: COX/SDH staining and ddPCR plots. (**A**) Longitudinal biopsy section. (**B**) Transverse biopsy section with COX negative cell labelled as 1. (**C**) RBF cell labelled as 2. (**D**) Intermediate cell labelled as 3 and COX positive cell labelled as 4. (**E**) Example of ddPCR 1D plot where each droplet from a sample is plotted on the graph of fluorescence intensity vs. droplet number. All positive droplets, blue for FAM or green for HEX, are scored as positive and each is assigned a value of 1. All negative droplets (grey), are scored as negative. (**F**) The histogram which plots amplitude vs. the frequency of the populations of droplets.

Open quadriceps muscle biopsies were taken under local anesthesia from all patients as well as from controls. All muscle biopsies were frozen in liquid nitrogen-cooled isopentane and stored at −80 °C until processed. Adjacent 10-µm sections were cut using a cryostat microtome and mounted on PEN membrane glass slides (Thermofisher). In this study, we used both transversal and longitudinal sections of the muscle biopsy (Fig. 1A,B). We were successful in obtaining longitudinal sections from only three of the muscle biopsies (patients 1, 3 and 5), due to the known difficulties in cutting sections from frozen tissue through long segments of individual muscle fibers^21^.

### Histochemical analysis and classification of individual muscle fibers

To highlight the muscle fiber segments deficient for OXPHOS, sequential Cytochrome c Oxidase (COX)/succinate dehydrogenase (SDH) histochemistry was carried out on each section in the same incubation. COX is partially encoded by mtDNA (COXI, II and III subunits), thus COX activity becomes deficient if mtDNA deletion crosses a certain threshold of heteroplasmy. SDH, on the contrary, is completely encoded by nuclear DNA, thus its enzymatic activity persists even in the presence of high loads of mtDNA deletion, or when a strong compensatory mitochondrial biogenesis occurs in the so-called ragged-blue-fibers (RBF)^22^. In fact, the RBF fibers are characterized by a massive accumulation of abnormal mitochondria that is visually distinctive compared to the other COX negative cells, as evidenced by a deep blue SDH reaction equivalent to the ragged red fibers. COX activity was demonstrated histochemically in a medium containing 4 mM 3,3′- diaminobenzidine tetrahydrochloride (DAB) and 100 mM cytochrome c (Sigma) in 0.1 M phosphate buffer, pH 7.0 at 37 °C. SDH activity was assayed by using 1.5 mM nitrobluetetrazolium, 130 mM sodium succinate, 0.2 mM phenazine methosulfate, and 1.0 mM sodium azide in 0.1 M phosphate, pH 7.0 at 37 °C. COX activity was detected after 40-min incubation, allowing the detection of histochemically COX positive muscle tissue. The incubation time for the SDH reaction was 30 min, allowing the detection of SDH activity, which was prevalent or exclusive in COX negative/SDH positive fibers^23^.

According to the COX/SDH staining muscle fibers were classified as COX positive (brown), COX intermediate (grey), COX negative (blue), RBFs (intense blue) (representative images are reported in Fig. 1B–D, all sections are reported in Fig. S1).

### Laser Capture Microdissection

The Leica laser capture microdissection (LCM) system (Leica Biosystems) was used, following manufacturer’s instructions to isolate 12-µm thick COX positive, COX intermediate, COX negative and RBF regions of single fibers, in both transversal and longitudinal sections. Prior to collection, each fiber was numbered and the total surface of the fiber was measured. This system uses UV laser and gravity collection. Presence of the single fiber in the collection tubes was always verified. Before microdissection, the tissue sections were subjected to an ethanol and dehydration series for 10–15 min at each step.

### Lysis of single cells for DNA amplification

Laser dissected cells were lysed in 2.5 ul of lysis buffer containing: 50 mM Tris-HCl pH 8.5, 200 ng/ml of Proteinase K (Invitrogen) and 1% Tween (Sigma). Cells were incubated for 16 hours at 55 °C and then 10 minutes at 95 °C for denaturation of Proteinase K. After the lysis, cells were digested for 1 hour at 37 °C with enzyme *PvuII* (Thermo Fisher Scientific) following the manufacturer’s instruction.

### Digital droplet PCR of DNA extracted from single cells

After the lysis and digestion, mtDNA deletion levels were analyzed and detected using the duplex *MTND1/MTND4* method. Digital PCR analysis was performed according to the published protocol established on DNA derived from muscle homogenates with certain modifications due to the different ddPCR platform used^18^. The limited amount of available DNA from single cells did not allow DNA quantification prior to analysis. Primer and probe sequences are shown in Table 2. (Primer numbers refer to the mtDNA Genbank reference sequence NC 012920.1).

**Table 2:** Primers and probes used in ddPCR experiments.

ND1 Forward	5′-CCCTAAAACCCGCCACATCT-3′
ND1 Reverse	5′-GAGCGATGGTGAGAGCTAAGGT-3′
ND1 Probe	5′-FAM/CCATCACCCTCTACATCACCGCCC/BHQ1-3′
ND4 Forward	5′-CCATTCTCCTCCTATCCCTCAAC-3′
ND4 Reverse	5′-CACAATCTGATGTTTTGGTTAAACTATATTT-3′
ND4 Probe	5′-HEX/CCGACATCATTACCGGGTTTTCCTCTTG/BHQ2-3′

Primer numbers refer to the mtDNA Genbank reference sequence NC 012920.1.

The 20 ul reactions consisted of 1X digital PCR supermix for probes (Bio-Rad Laboratories), 375 nM of ND1 probe, 900 nM of ND1 primers, 125 nM of ND4 probe, 450 nM ND4 primers and nuclease-free water. Reactions were transferred to 96-well plates (Eppendorf) and droplet generation was carried out in an Automated Droplet Generator with DG32 cartridges (Bio-Rad Laboratories). Automated Droplet Generator partitions samples into around 20,000 nanoliter-sized droplets. Droplets are automatically transferred to 96-well plates ready for further amplification. PCR reactions were performed on a C1000 Touch thermal cycler (Bio-Rad Laboratories) using standard cycling conditions as follows: 95 °C-10 minutes, 40 cycles of 94 °C-30 seconds, 40 cycles of 60 °C-1 minute and 98 °C- 10 minutes.

Following PCR amplification of the nucleic acid target, droplets were analyzed on a QX200 Droplet Digital PCR (ddPCR™). Each droplet was analyzed individually using a two-color detection system (set to detect FAM and HEX); PCR-positive and PCR-negative droplets are counted to provide absolute quantification of target DNA in digital form using QuantaSoft™Pro software (Bio-Rad). Results are collected and visually examined in 1D and 2D plots as represented in Fig. 1E. The level of mtDNA wild type molecules is calculated by dividing the absolute number of *MTND4* with *MTND1* and the percentage of mtDNA deletion is 1-wt molecules^24^. The mtDNA copy number density was measured at the single fiber level and presents the total number of mtDNA molecules in a muscle fiber section, divided by the fiber’s cross-sectional area^25^ . Each sample was assessed in triplicate.

### Single cell long range PCR

Long range PCR was carried out using PrimeSTAR GXL DNA polymerase (TaKaRa, Clontech) on available DNA samples extracted from individual muscle fibers. Briefly, single muscle fibers were laser dissected, lysed and digested as described earlier. Due to limited amounts of DNA, quantification prior to PCR was not possible. Primer pairs were: forward 6222–6240 and reverse 16133–16153. Reaction was prepared as follows: 1× buffer, 0.2 uMdNTP mix, 0.4 uM forward and reverse primers, 0.625 unit polymerase and 3 ul DNA. Cycling conditions were: 94 °C-1 minute; 35 cycles of 94 °C-10 seconds, 60 °C-15 seconds and 68 °C-10 minutes; 72 °C-10 minutes. Amplified PCR products were separated through 0.7% agarose gels. Smaller bands, suggesting the presence of mtDNA deletions were cut out of gels. DNA was extracted according to manufacturer’s protocol with QIAquick Gel Extraction Kit (Qiagen) and sequenced on an ABI3130xl using BigDye terminator v3.1.

### Statistical analysis

For the purpose of group comparisons, ANOVA statistical analysis was performed using GraphPad™ v.5 statistical software (GraphPad Software). Group comparisons were considered to be statistically non-significant (ns) if the calculated P-value was greater than 0.05, significant (*) with P = 0.01 to 0.05, very significant (**) with P = 0.001 to 0.01 and extremely significant (***) when the P-value was less than 0.001.

### Data availability statement

The datasets generated during and/or analysed during the current study are available from the corresponding author on reasonable request.

## Results

### Validation of ddPCR by comparison with Southern blot

**Figure 2:**
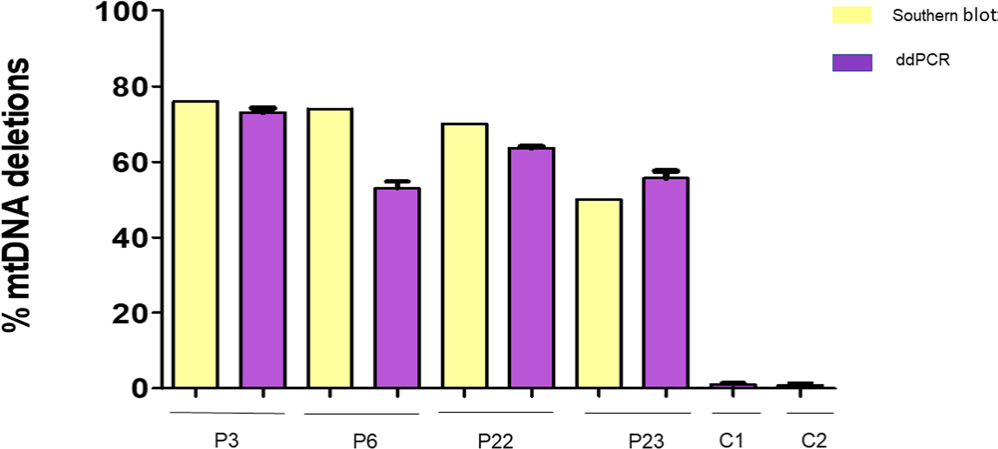
Comparison between ddPCR and Southern blot results on muscle homogenate or blood DNA. Southern blot indicated mtDNA deletion levels of 76.0% and 74.0% in DNA extracted from muscle homogenates of patients 3 and 6, respectively. Mean values from ddPCR were 73.1% (SD = 2.3) and 53.0% (SD = 4), respectively. In patients 22 and 23, heteroplasmy rate of mtDNA deletions on Southern blot in blood derived DNA was 70.0% and 50.0%, while ddPCR estimated 63.7% (SD = 0.58) and 55.7% (SD = 3.2), respectively. Heteroplasmy rate of mtDNA deletions in muscle homogenate DNA from controls 1 and 2 validated by ddPCR was 1.0% (SD = 0.8) and 0.9% (SD = 0.8), respectively. All ddPCR experiments were performed in triplicates. The SD values are presented as error bars.

To validate the ddPCR method, we used homogenate muscle DNA extracted from muscle biopsies of patients carrying single or multiple mtDNA deletions that had previously been identified by Southern blot. A major advantage of ddPCR is its high sensitivity and consequently, the amount of DNA required is significantly lower compared with that needed for Southern blotting. We detected similar heteroplasmy loads with these two methods (Fig. 2). As a drawback, the size of the deletion is not assessed by ddPCR, and can only be estimated with good approximation by Southern blot. As we found that ddPCR is much more sensitive than Southern blot in detecting and quantifying mtDNA deletions, we also tested whether it detects single mtDNA deletions in blood cells from patients with the Pearson and Kearns Sayre syndromes or CPEO (n = 16). Single mtDNA deletions had previously been found in homogenate muscle DNA by long range PCR and/or Southern blot. Results of ddPCR and Southern blot with heteroplasmy estimation as well as long-range PCR results for these samples are summarized in Table S1. We could only detect single mtDNA deletions in blood cells of two children with clinical presentation of Pearson syndrome (<1 year old) and Kearns-Sayre Syndrome (16 years old). In both children, the deletion was also detectable on LR-PCR. In blood DNA of adult patients with CPEO, ddPCR failed to detect the single mtDNA deletion.

### COX/SDH activities define the pattern of OXPHOS impairment at the single fiber level

Histochemical staining of muscle biopsy sections is an established method of demonstrating OXPHOS impairment and four subpopulations of muscle cells can be distinguished: COX positive, COX intermediate, COX negative, and RBF (Fig. 1). Skeletal muscle is therefore the tissue of choice to investigate the clonal expansion of deleted mtDNA in single cell populations that exhibit different OXPHOS deficits. We investigated both single mtDNA deletions and multiple mtDNA deletions secondary to *POLG* and *OPA1* mutations. To fully explore the distribution of deleted mtDNA, we performed our quantitative assessment on both transverse and longitudinal biopsy sections. Overall, we investigated individual fibers collected by laser capture micro-dissection in 7 patients and 3 controls: COX positive (n = 265), COX negative (n = 70), intermediate (n = 98) and RBF (n = 52) (Fig. 1A–D and Fig. S1).

COX negative fibers were absent in the control muscle specimens. The lowest percentages of COX negative fibers were found in the biopsies from Patients 1 and 2, who both carried a heterozygous dominant *OPA1* mutation, with levels of 2.5% and 5%, respectively. The highest percentages of COX negative fibers were found in the muscle of Patient 5 (28.5%) who carried a single “common” deletion, and in Patient 3 (28.3%) who carried recessive *POLG* mutations. The percentages of COX negative fibers in Patient 3 also included the RBFs. The percentage of intermediate fibers was lowest in Patients 1 and 2, being 2% and 4.3%, respectively. The highest load of intermediate fibers was found in the biopsy from Patient 3 (20.7%) and Patient 6 (18.5%), who carried a single “common” deletion (Table 3).

**Table 3:** Proportion of COX negative fibers, RBFs and intermediate fibers.

Case	Genetic defect	%COX negative (including RBF’s)	%COX intermediate
P1	*OPA1*	2.53	2
P 2	*OPA1*	5	4.3
P 3	*POLG*	28.3	20.7
P 4	*POLG*	12.5	16
P 5	SINGLE DELETION	28.5	16.5
P 6	SINGLE DELETION	3.94	18.5
P 7	SINGLE DELETION	10.8	20

### Levels of mtDNA deletions in two patients with OPA1 mutations and DOA “plus”

**Figure 3:**
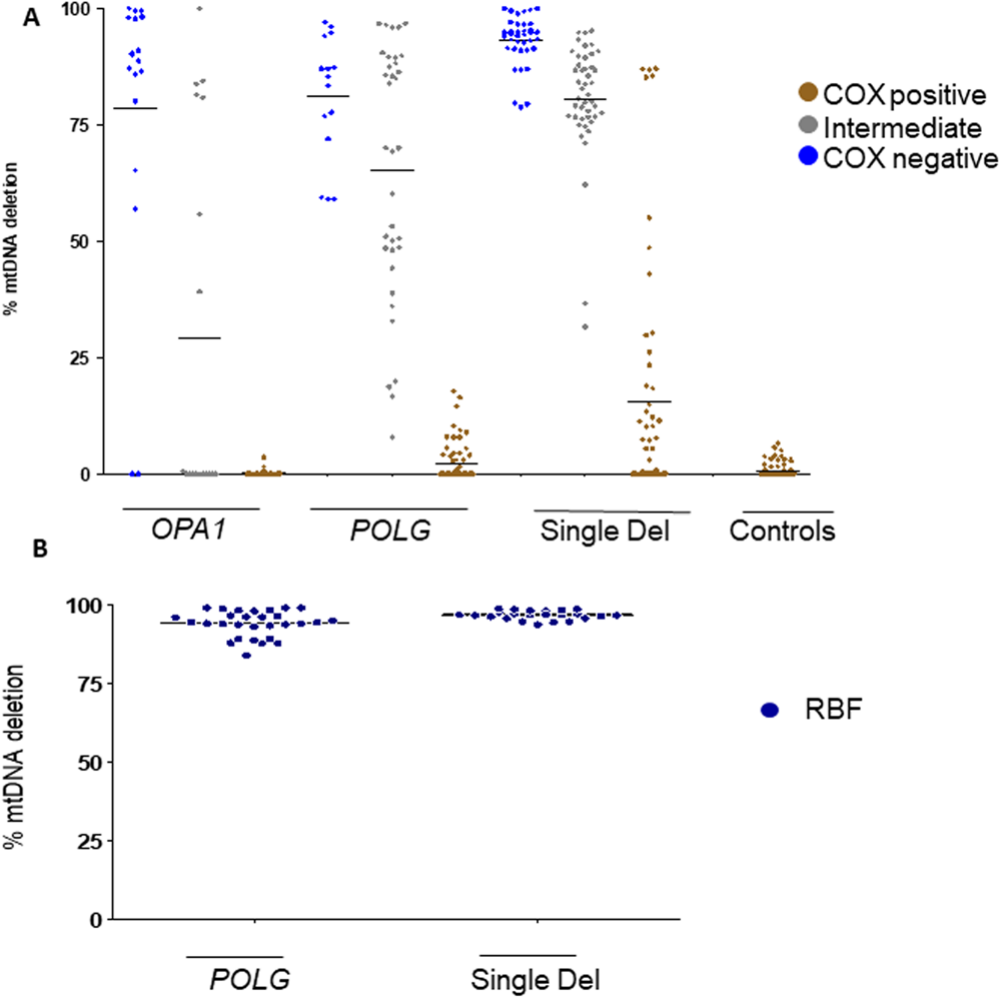
Level of mtDNA deletions obtained with ddPCR in single muscle fibers. (**A**) COX positive (brown dots), COX negative (light blue dots) and intermediate fibres (grey dots). The groups represent pooled data of fibres from different patients *OPA1* (P1 and P2); *POLG* (P3 and P4); Single deletion (P5, P6 and P7); Control (C1, C2 and C3). All measurements were performed in triplicates. The mean values for each group of fibres are presented with black horizontal lines. (**B**) Level of mtDNA deletions obtained with ddPCR in RBF fibres. These fibers were present only in the *POLG* and single deletion groups.

We assessed the mtDNA deletion load by ddPCR in COX negative fibers (n = 17), intermediate fibers (n = 17) and COX positive fibers (n = 27) dissected by LCM from muscle biopsies of Patients 1 and 2 (Fig. 3). The mean value of deletion load in COX negative fibers was 78.5% (SD = 30.8). Deletion load was lower in intermediate fibers, with a mean value of 29.2% (SD = 39). In COX positive fibers from the *OPA1* group, mtDNA deletions were hardly detected, with a mean deletion load of 0.3% (SD = 0.8). Deletions were absent in two of the COX negative fibers from Patient 1. This value was not different from that assessed in COX positive fibers from controls (n = 105), with a mean of 0.5% (SD = 1.3) (Fig. 3).

### Levels of mtDNA deletions in two patients with autosomal recessive POLG mutations and SANDO

The mean mtDNA deletion load in COX negative fibers (n = 15) from Patients 3 and 4 was 81% (SD = 13.3). We also analyzed separately the subcategory of COX negative fibers defined as RBFs (n = 29). These fibers expressed the highest levels of mtDNA deletions, with a mean value of deleted molecules reaching 94.1% (SD = 4.1) (Fig. 3). Intermediate fibers (n = 35) harbored a mean deletion load of 65 % (SD = 27.07). COX positive fibers (n = 69) had a limited load of deleted molecules, reaching the mean value of 2.1% (SD = 4), not statistically different from controls (Fig. 3).

### Levels of the single mtDNA deletion in three patients with CPEO

The mean deletion load in COX negative fibers (n = 37) of Patients 5, 6 and 7 was 93.1 % (SD = 5.4) compared with a mean value of 96.8% (SD = 1.4) for RBFs (n = 23). Deletion load in intermediate cells had a mean value of 80.5% (SD = 12.5). Remarkably, more than 50% (27 out of 55 COX positive fibers) harbored variable percentages of mtDNA deletion, reaching a mean value of 15.8% (SD = 25) (Fig. 3).

### Different profiles of mtDNA deletion accumulation amongst the patient groups

The mtDNA deletion levels in COX negative fibers from single deletion patients were significantly higher (P < 0.05*), with a mean value of 93.08% compared with 78.5% and 81% in *OPA1* and *POLG* patients, respectively. However, there were no significant differences of deletion levels in COX negative cells between *OPA1* and *POLG* patients. RBFs were only observed in the *POLG* and single deletion groups, and significantly higher percentages were present in the single deletion group compared with the POLG group (*P < 0.05).

The intermediate fibers still retain a partial COX activity, approaching the threshold level to turning into COX negative. Our results showed that this threshold might be different at the single fiber level. In fact, the load of mtDNA deletions in single intermediate fibers varied in each patient group, from a mean value of 29.2% in the *OPA1* patients, 65% in *POLG* patients and 80.5% in single deletion patients. Again, the patterns of mtDNA deletion accumulation were significantly different (***p < 0.0001) when the three groups were compared.

As COX-positive fibers are considered to be fully functional with normal OXPHOS activity, these cells are assumed to generally have either no mtDNA deletions or very low amounts, below a certain threshold^25,26^. Our ddPCR results, obtained from a large number of COX-positive fibers (n = 265) collected from patients and controls, showed that COX-positive cells from single deletion patients had a significantly larger accumulation of mtDNA deletions when compared to both *POLG* and *OPA1* patients, and with the control group (***P < 0.0001) (Fig. 3).

### Levels of mtDNA deletions in longitudinal muscle biopsy sections

All previous assessments of mtDNA deletion loads were performed in single fibers dissected by laser capturing from a cross-sectional profile of the fiber in transverse sections of the muscle biopsy. Thus, each evaluation refers to distinct cells. However, muscle fibers viewed in longitudinal section may display COX-positive domains intermixed with COX-intermediate/COX-negative domains^21^. Thus, within the same muscle fiber the mtDNA deleted molecules are accumulated by clonal expansion in specific domains. To explore this feature in our samples we applied LCM to longitudinal sections obtained from the muscle biopsies of patients 1, 3 and 5, representative of each pathological group. COX-positive domains were analyzed and compared with COX-negative domains from the same fibers in each group.

**Figure 4:**
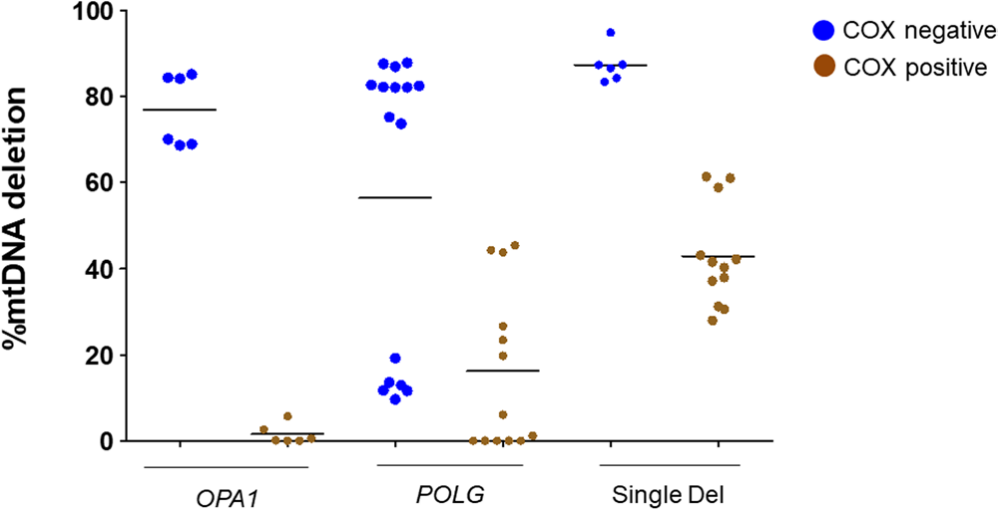
Level of mtDNA deletions in longitudinal fibres obtained by ddPCR. The highest load of mtDNA deletions was detected in the COX negative fiber segments (blue dots) from the single deletion group. All measurements were performed in triplicates. Longitudinal fibers were analyzed from the following patients: *OPA1* group (P1); *POLG* (P3); single deletion group (P5). The mean values for each group of fibers are presented with black horizontal lines.

This analysis confirmed that the single deletion group had the highest levels of mtDNA deletion in the COX negative domains of individual fibers (mean = 87.32%, SD = 4.06) as compared with the *OPA1* (mean = 76.88%, SD = 8.43) and *POLG* (mean = 56.34%, SD = 34.82) groups. Similarly, the assessment in COX positive domains of the same individual fibers paralleled the previous results from muscle cross-sections, with the single deletion group having the highest load of mtDNA deleted molecules (mean=42.8%, SD = 11.7) compared with the *OPA1* (mean = 1.5%, SD = 2.3) and *POLG* (mean = 16.2%, SD = 18.7) groups (Fig. 4).

Qualitatively, more defined areas of COX negativity were visualized in *POLG* and *OPA1* longitudinal sections of muscle biopsies. In comparison, longitudinal fibers from the single deletion case had a wider dispersion of COX negativity. In fact, it was challenging to select fibers from the single deletion case that contained well defined COX negative and COX positive zones, due to frequent areas of blue staining invading the entire length of the fiber in a stripe-like fashion. We also sampled those fibers that appeared entirely COX positive along their longitudinal length (Fig. S2). In this latter case, we found a significant amount of mtDNA deletion in two fully COX positive longitudinal fibers (fiber 1: mean 30%; fiber 2: mean 60.4%), highlighting the hallmark of the single deletion cases that had widespread amounts of mtDNA deletion affecting fibers with apparently functional OXPHOS.

To further confirm our ddPCR findings, we used long-range PCR to amplify DNA extracted from single longitudinal fibers. Long-range PCR is not a quantitative method as it preferentially amplifies the shorter, deleted mtDNA molecules, thus it is not used for accurate determination of the heteroplasmy of deletions. However, it was useful to confirm the presence of deleted mtDNA molecules in COX positive longitudinal fibers from single deletion patient by this approach (Fig. S3).

### MtDNA copy number assessment reveals compensatory mtDNA proliferation in muscle fibers with impaired OXPHOS

**Figure 5:**
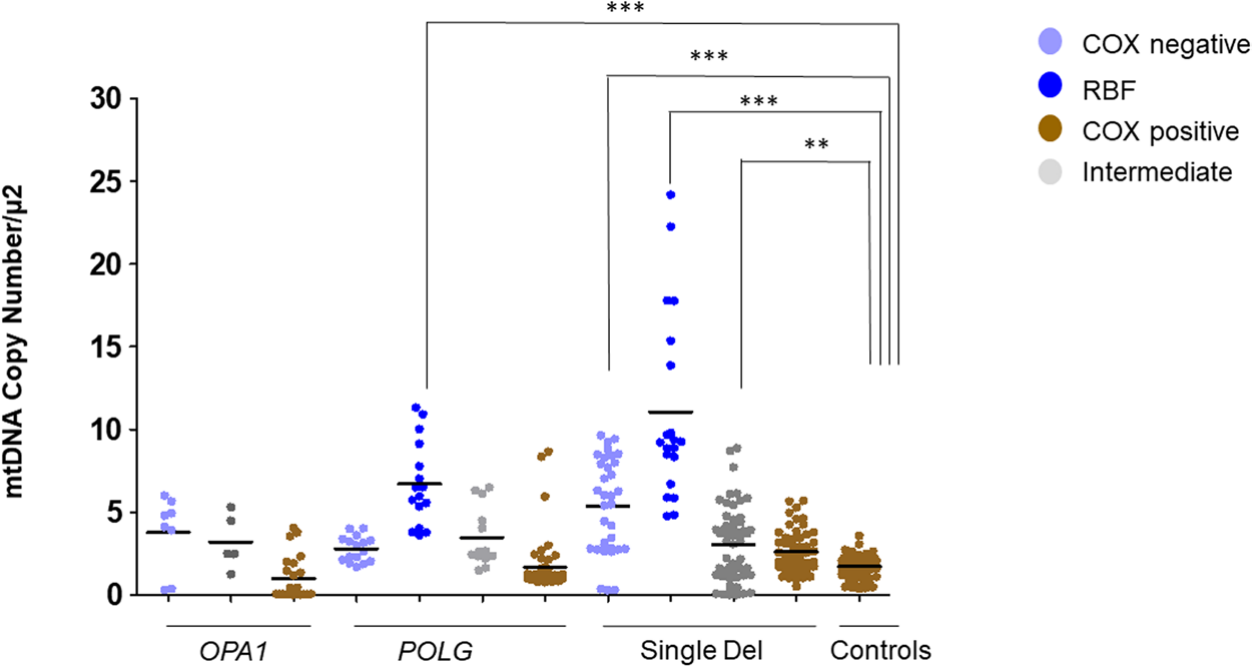
MtDNA copy number density assessed by ddPCR. The groups represent pooled data of fibres from different patients; *OPA1* group (P1 and P2); *POLG* group (P3 and P4); single deletion group (P5, P6 and P7); control group (C1, C2 and C3). All measurements were performed in triplicates. The mean values for each group are presented with black horizontal lines. Levels of significance: **P = 0.001–0.01; ***P < 0.001.

We compared total mtDNA copy number per um^2^ between the different subcategories of fibers in patients and controls. MtDNA copy number was variable in the control group, ranging from 0.4 to 3.6 copies/um^2^ with a mean value of 1.7 (SD = 0.7). This variability is possibly due to differences in type 1 and 2 fibers ratio, and to the variable level of physical activity^27^. Our results revealed that COX negative, RBFs and intermediate fibers from the single deletion group had a significant increase in mtDNA density compared with healthy controls, similar to the RBFs from the *POLG* group. The deletion load was higher in fibers with high mtDNA copy numbers. COX positive fibers from all groups, as well as COX negative and intermediate from *POLG* and *OPA1* group, did show increased values compared to controls, failing to achieve statistical significance (Fig. 5).

## Discussion

We have shown that ddPCR is a sensitive and reliable assay to quantify the level of single or multiple mtDNA deletions in individual muscle fibers. Three groups of patients with mitochondrial disease were assessed (single large-scale mtDNA deletion and multiple mtDNA deletions due to pathogenic *OPA1* and *POLG* mutations) in single muscle fibers laser captured in transverse and longitudinal sections of muscle biopsies. Different domains of the muscle fibers were considered on the basis of the histochemical double staining for COX/SDH activities. This analysis revealed different profiles of mtDNA deletion abundance and distribution that likely result from different underlying mechanisms and timing of clonal expansion of the deleted molecules.

The patients with single mtDNA deletions had variable amounts of deleted molecules even in the COX positive fibers, indicating a stochastic distribution of the mtDNA deletion, which presumably occurred in the early stages of embryonic development or clonally expanded from a pre-existing pool in the oocyte. In contrast, the patients affected with DOA “plus” and carrying dominant *OPA1* mutations leading to defective mitochondrial fusion had extremely low amounts of deleted mtDNA molecules in the COX positive fibers, comparable to controls. This indicates that the fusion defect favored over lifespan the clonal expansion of pre-existing mtDNA deletions, possibly those normally accumulated with ageing, but only in specific domains of the muscle fibers. This might be due to the fusion defect that limited the clonal expansion along the length of the fiber, keeping the COX negative domain restricted. Finally, the patients affected by SANDO syndrome, due to recessive *POLG* mutations leading to defective mtDNA replication, had increased amounts of deleted mtDNA molecules in the COX positive fibers, intermediate between controls/*OPA1* patients and single deletion patients. This would suggest that mtDNA multiple deletions accumulate at accelerated rate somatically over the lifespan of patients, with increased rate of clonal expansion in specific domains of the muscle fibers that might expand in the length of the fiber over time.

Noticeably, we assessed mtDNA copy number, as a surrogate measure of activated mitochondrial biogenesis in the same single cells we used for assessing the loads of mtDNA deleted molecules. The results provided evidence that where the deletion load was highest, higher mtDNA copy number was also recognized. In other words, the COX negative fibers in single deletion cases and the RBFs in both single deletion and *POLG* had the highest mtDNA copy number, demonstrating the strongest attempt to compensate for OXPHOS deficiency by compensatory activation of mitochondrial biogenesis. Thus, these results provide compelling evidence that if the mtDNA deletion crosses a certain threshold to trigger compensatory mitochondrial biogenesis and mtDNA replication, the deleted molecules take advantage for their own numeric expansion^28^. The mtDNA copy number is tightly regulated and dependent on the balanced processes of mitochondrial biogenesis and clearance by mitophagy, which maintain the mitochondrial network mass through highly regulated cycles of fusion and fission of organelles^16^. Recent studies on *Caernorhabditis elegans* (*C. elegans*) strains carrying a single mtDNA deletion provided evidence that the deleted molecules highjack the activation of the mitochondrial unfolded protein response (mtUPR) to clonally expand, implicating either reduced mitophagy^29^ or activated mitochondrial biogenesis and organelle dynamics^30^. Interestingly, Gitschlang *et al*. investigated the issue of a possible replicative advantage of the deleted mtDNA molecules, due to their shorter length^31,32^ failing to find any evidence that this actually occurs in *C. elegans*^29^. Similarly, a study addressing the same issue in human skeletal muscle also found no appreciable differences in the rate of clonal expansion between mtDNA deletions of different sizes^33^.

The mechanisms of reduced mitophagy or activated mitochondrial biogenesis and dynamics are not mutually exclusive, and they have all been implicated in both physiological and pathological states in skeletal muscle, the tissue used in the current study. The investigation of murine animal models with impaired fusion, as obtained by conditional deletions of both Mitofusins Mfn1 and Mfn2, has previously shown that mitochondrial dynamics is crucial for mtDNA maintenance^34^. More recently, the rate of fusion in single muscle fibers has been linked to specific and discrete mitochondrial domains correlated with oxidative capacity^35^. Thus, the local impairment of OXPHOS may unbalance mitochondrial dynamics and the relative rates of mitophagy and mitochondrial biogenesis, triggering a complex series of tightly connected events that may ultimately lead to the clonal expansion of deleted mtDNA molecules, which is remarkably restricted to these mitochondrial domains. However, in a recent study utilizing a *Drosophila Melanogaster* model, the loads of a heteroplasmic mtDNA deletion were reduced and the pathological phenotype ameliorated by stimulation of autophagy, activation of the PINK1/Parkin pathway or decreasing the levels of Mitofusins, all strategies aimed at limiting mitochondrial fragments to fuse again with the network and, thus, promoting their mitophagic elimination^36^. The complex role that mitochondrial dynamics plays has also been modeled mathematically showing that a higher rate of fusion-fission can provide faster clearance of mutant mtDNA, only when transiently existing mutant-rich mitochondria are efficiently prevented from re-fusing with other mitochondria and selectively removed^37^. Otherwise, faster fusion-fission accelerates the accumulation of mutant mtDNA^38–40^.

The different profiles of distribution and loads of deleted mtDNA molecules found by our study in the different categories of muscle fibers, in particular in the COX positive fibers, possibly reflect these purported mechanisms currently proposed for clonal expansion of deleted mtDNA.

In conclusion, our results shed new insight on the complex interrelated mechanisms underlying the clonal expansion of mtDNA deletions, crucially indicating different profiles dependent on the specific genetic defect and, possibly, on the time points of the original mutational event.

## Electronic supplementary material

Supplementary information

## References

[1] Giles RE, Blanc H, Cann HM, Wallace DC (1980). Maternal inheritance of human mitochondrial DNA. Proc. Natl. Acad. Sci. USA.

[2] Carelli V, Chan DC (2014). Mitochondrial DNA: impacting central and peripheral nervous systems. Neuron.

[3] Holt IJ, Harding AE, Morgan-Hughes JA (1988). Deletions of muscle mitochondrial DNA in patients with mitochondrial myopathies. Nature.

[4] Wallace DC (1988). Mitochondrial DNA mutation associated with Leber's hereditary optic neuropathy. Science.

[5] Zeviani M (1988). Deletions of mitochondrial DNA in Kearns-Sayre syndrome. Neurology.

[6] Zeviani M (1989). An autosomal dominant disorder with multiple deletions of mitochondrial DNA starting at the D-loop region. Nature.

[7] Pitceathly RD, Rahman S, Hanna MG (2012). Single deletions in mitochondrial DNA–molecular mechanisms and disease phenotypes in clinical practice. Neuromuscul. Disord..

[8] Chen X (1995). Rearranged mitochondrial genomes are present in human oocytes. Am. J. Hum. Genet..

[9] Brenner CA (1998). Mitochondrial DNA deletion in human oocytes and embryos. Mol. Hum. Reprod.

[10] Chinnery PF (2004). Risk of developing a mitochondrial DNA deletion disorder. Lancet..

[11] Poulton J, Deadman ME, Ramacharan S, Gardiner RM (1991). Germ-line deletions of mtDNA in mitochondrial myopathy. Am. J. Hum. Genet..

[12] Bernes SM (1993). Identical mitochondrial DNA deletion in mother with progressive external ophthalmoplegia and son with Pearson marrow-pancreas syndrome. J. Pediatr..

[13] Shanske S (2002). Identical mitochondrial DNA deletion in a woman with ocular myopathy and in her son with pearson syndrome. Am. J. Hum. Genet..

[14] Blakely EL (2004). Mitochondrial DNA deletion in "identical" twin brothers. J. Med. Genet..

[15] Viscomi C, Zeviani M (2017). MtDNA-maintenance defects: syndromes and genes. J. Inherit. Metab. Dis..

[16] Carelli V (2015). Mitochondria: Biogenesis and mitophagy balance in segregation and clonal expansion of mitochondrial DNA mutations. Int. J. Biochem. Cell Biol..

[17] Manoj P. Droplet digital PCR technology promises new applications and research areas. Mitochondrial DNA A DNA Mapp Seq. Anal. **27**(1), 742–746. (2016). 10.3109/19401736.2014.913168 24779593

[18] Belmonte FR (2016). Digital PCR methods improve detection sensitivity and measurement precision of low abundance mtDNA deletions. Sci. Rep..

[19] Carelli V (2015). Syndromic parkinsonism and dementia associated with OPA1 missense mutations. Ann Neurol..

[20] Amati-Bonneau P (2008). OPA1 mutations induce mitochondrial DNA instability and optic atrophy 'plus' phenotypes. Brain..

[21] Elson JL, Samuels DC, Johnson MA, Turnbull DM, Chinnery PF (2002). The length of cytochrome c oxidase-negative segments in muscle fibres in patients with mtDNA myopathy. Neuromuscul. Disord..

[22] Reichmann H, Vogler L, Seibel P (1996). Ragged red or ragged blue fibers. Eur. Neurol..

[23] Old SL, Johnson MA (1989). Methods of microphotometric assay of succinate dehydrogenase and cytochrome c oxidase activities for use on human skeletal muscle. Histochem. J..

[24] He L (2002). Detection and quantification of mitochondrial DNA deletions in individual cells by real-time PCR. Nucleic Acids Res.

[25] Yu-Wai-Man P (2010). OPA1 mutations cause cytochrome c oxidase deficiency due to loss of wild-type mtDNA molecules. Hum. Mol. Genet.

[26] Bua E (2006). Mitochondrial DNA-deletion mutations accumulate intracellularly to detrimental levels in aged human skeletal muscle fibers. Am. J. Hum. Genet..

[27] Adhihetty PJ, Taivassalo T, Haller RG, Walkinshaw DR, Hood DA (2007). The effect of training on the expression of mitochondrial biogenesis- and apoptosis-related proteins in skeletal muscle of patients with mtDNA defects. Am. J. Physiol. Endocrinol. Metab..

[28] Herbst A, Johnson CJ, Hynes K, McKenzie D, Aiken JM (2013). Mitochondrial biogenesis drives a vicious cycle of metabolic insufficiency and mitochondrial DNA deletion mutation accumulation in aged rat skeletal muscle fibers. PLoS One..

[29] Gitschlag BL (2016). Homeostatic responses regulate selfish mitochondrial genome dynamics in C. elegans. Cell Metab..

[30] Lin YF (2016). Maintenance and propagation of a deleterious mitochondrial genome by the mitochondrial unfolded protein response. Nature..

[31] Wallace DC (1992). Mitochondrial genetics: a paradigm for aging and degenerative diseases?. Science..

[32] Diaz F (2002). Human mitochondrial DNA with large deletions repopulates organelles faster than full-length genomes under relaxed copy number control. Nucleic Acids Res.

[33] Campbell G, Krishnan KJ, Deschauer M, Taylor RW, Turnbull DM (2014). Dissecting the mechanisms underlying the accumulation of mitochondrial DNA deletions in human skeletal muscle. Hum. Mol. Genet..

[34] Chen H (2010). Mitochondrial fusion is required for mtDNA stability in skeletal muscle and tolerance of mtDNA mutations. Cell..

[35] Mishra P, Varuzhanyan G, Pham AH, Chan DC (2015). Mitochondrial dynamics is a distinguishing feature of skeletal muscle fiber types and regulates organellar compartmentalization. Cell Metab..

[36] Kandul NP, Zhang T, Hay BA, Guo M (2016). Selective removal of deletion-bearing mitochondrial DNA in heteroplasmic Drosophila. Nat. Commun..

[37] Tam ZY, Gruber J, Halliwell B, Gunawan R (2015). Context-Dependent Role of Mitochondrial Fusion-Fission in Clonal Expansion of mtDNA Mutations. PLoS Comput. Biol..

[38] Iommarini L (2012). Revisiting the issue of mitochondrial DNA content in optic mitochondriopathies. Neurology..

[39] Sitarz KS (2012). OPA1 mutations induce mtDNA proliferation in leukocytes of patients with dominant optic atrophy. Neurology..

[40] Liao C (2017). Dysregulated mitophagy and mitochondrial organization in optic atrophy due to OPA1 mutations. Neurology.

