# Supplementary material for: Clonal expansion of mtDNA deletions: different disease models assessed by digital droplet PCR in single muscle cells
Source: Sci Rep. 2018 Aug 3;8:11682. doi: 10.1038/s41598-018-30143-z (PMC6076247; doi:10.1038/s41598-018-30143-z)
Supplement: Supplementary file 1 — Supplementary information [file 41598_2018_30143_MOESM1_ESM.pdf]

Supporting information Note: Case Reports

Patient 1:

A 76-year-old man presented since he was young with the main symptoms of muscle weakness, gait unsteadiness, chronic and bilateral ptosis. Psychiatric symptoms included anxiety, panic attacks and depression. All these symptoms progressed and later in time he developed peripheral neuropathy, deafness, optic atrophy and Parkinsonism. At the time of biopsy he was 66 years old, and biopsy revealed cytochrome c oxidase (COX) negative fibres. The mtDNA analysis revealed the presence of multiple mtDNA deletions by long-range PCR. The patient belonged to a large family with dominant transmission of the same phenotype. Screening of candidate genes involved with multiple mtDNA deletions revealed a heterozygous missense mutation in OPA1, affecting nucleotide c.1462G>A. This mutation affects the conserved amino acid p.G488R in the GTPase domain of OPA1. This family has been previously reported (Carelli, 2015).

Patient 2:

This is a 49-year-old man who had poor vision since he was 4-year-old that progressed to complete blindness later in life. Since 9 years of age he also suffered a progressive hearing loss needing acoustic prosthesis. At 30 years of age he developed gait difficulties with frequent falls due to peripheral neuropathy. At the time of muscle biopsy he was 42, and biopsy was positive for COX negative fibres. Electron microscopy of skeletal muscle showed mitochondria with morphologically abnormal cristae and accumulation of lipid droplets. Long-range PCR analysis of mtDNA revealed the presence of mtDNA multiple deletions. Screening of candidate genes was positive for the mutation c.1316 G>T affecting a conserved amino acid p.G439V in the GTPase domain of OPA1. The patient's daughter developed the same phenotype and this case has been previously reported (Amati-Bonneau, 2008).

Patient 3:

This is a 63-year-old man with a multisystemic syndrome known with the acronym of SANDO. He suffered with ptosis and ophthalmoplegia by the age of 30; ten years later he started to develop a progressive skeletal muscle weakness and a sensitive polyneuropathy causing an ataxic gait. By the age of 50 he had dysarthria. His muscle biopsy showed a mitochondrial myopathy with Ragged Red Fibres and COX negative fibres. He carried two heterozygous mutations in the *POLG1* gene (a missense mutation c.934T>C causing the amino acid change p.W312R and an insertion, c.3629insA, causing a stop codon p.Y1210X). mtDNA multiple deletions were evident with long range PCR of muscle DNA.

Patient 4:

This 67-year-old woman suffered from ptosis and ophthalmoplegia from the age of 30 years. By the age of 56 years she clinically presented with ptosis, ophthalmoplegia, skeletal muscle weakness, peripheral polyneuropathy. A few years later she also developed dysphagia and a Parkinsonian syndrome with hypomimia, hypophonia, camptocormia and bradykinesia. Her muscle biopsy showed a mitochondrial myopathy with Ragged Red Fibres and COX negative fibres. She carried a homozygous missense mutation c.1943C>G in *POLG1* gene leading to the amino acid change p.P648R. Multiple mtDNA deletions were evident with long range PCR.

Patient 5:

This 50-year-old man started to develop a progressive bilateral ptosis and ophthalmoplegia by the age of 17 years. By the age of 45 years he suffered from diabetes mellitus, cardiomyopathy and skeletal muscle weakness. His muscle biopsy revealed numerous COX negative fibres. He carried a single mtDNA deletion of 4977 base pairs (nt. 8469-nt.13447).

Patient 6:

This 23-year-old girl suffered from monolateral ptosis by the age of 15 years without ophthalmoparesis. Her neurological examination was negative except for monolateral ptosis and electromyography didn’t show signs of myopathy and/or polyneuropathy. Audiometry was normal. She had lactic acidosis after aerobic effort and muscle biopsy showed a mitochondrial myopathy with COX negative fibres. She carried a single mtDNA deletion of 4977 base pairs (nt. 8469-nt.13447).

Patient 7: This 55-year-old man developed ophthalmoparesis by the age of 18 and later, at 40 years, a bilateral progressive ptosis; he also presented with a mild peripheral polyneuropathy and neurosensorial hearing loss. His muscle biopsy showed a mitochondrial myopathy with COX negative fibres, without Ragged Red fibres. He carried a single mitochondrial DNA deletion of 5992 base pairs (breakpoint nt.9431-nt.15423). At the time of the biopsy he was 50 years old.

Control 1: 37 year old female

Control 2: 20 year old female

Control 3: 41 year old female

Figure S1. Biopsy COX/SDH stain.

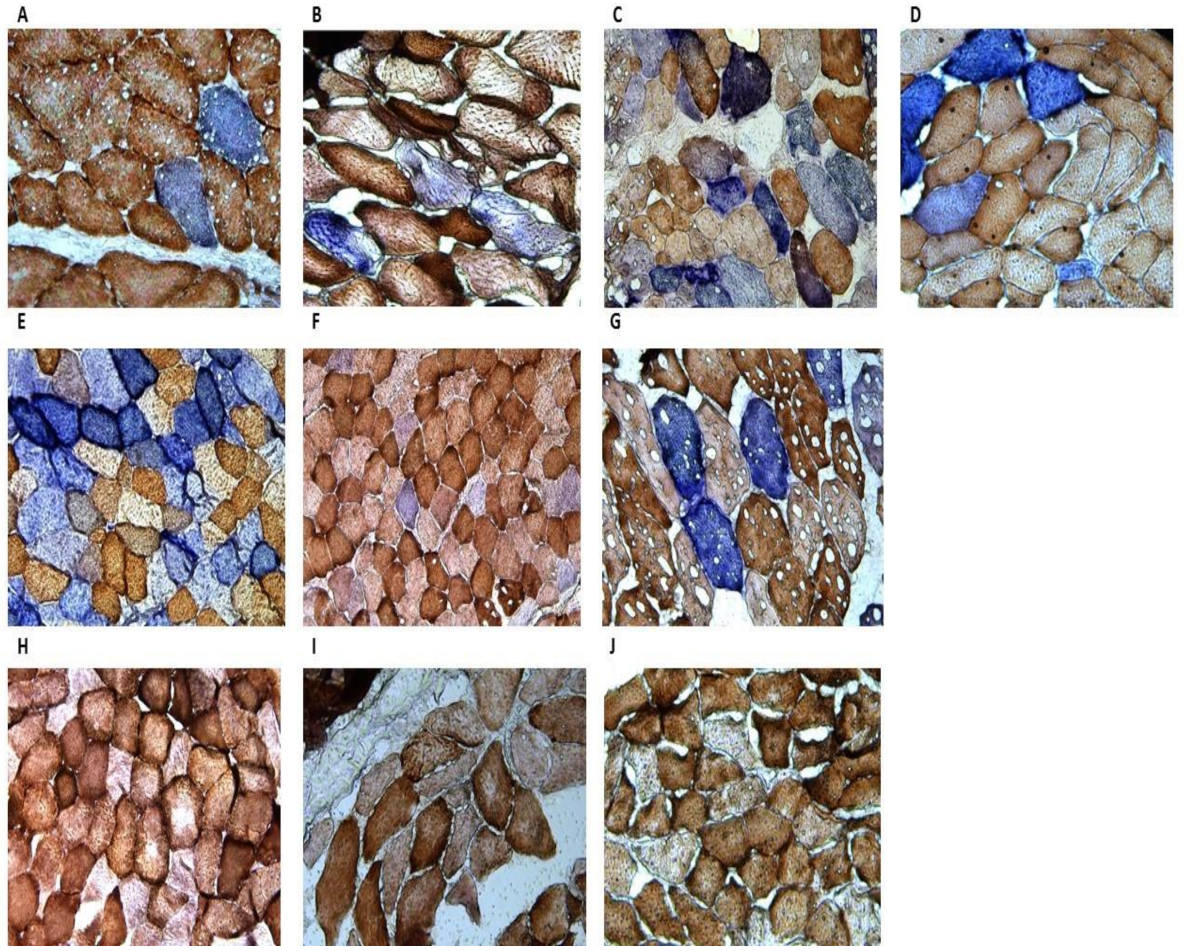

A-biopsy belonging to patient 1; B- biopsy belonging to patient 2; C- biopsy belonging to patient 3; D- biopsy belonging to patient 4; E- biopsy belonging to patient 5; F- biopsy belonging to patient 6; G- biopsy belonging to patient 7; H- biopsy belonging to control 1; I- biopsy belonging to control 2; J-biopsy belonging to control 3.

Figure S2. COX positive longitudinal fibers from patient 5.

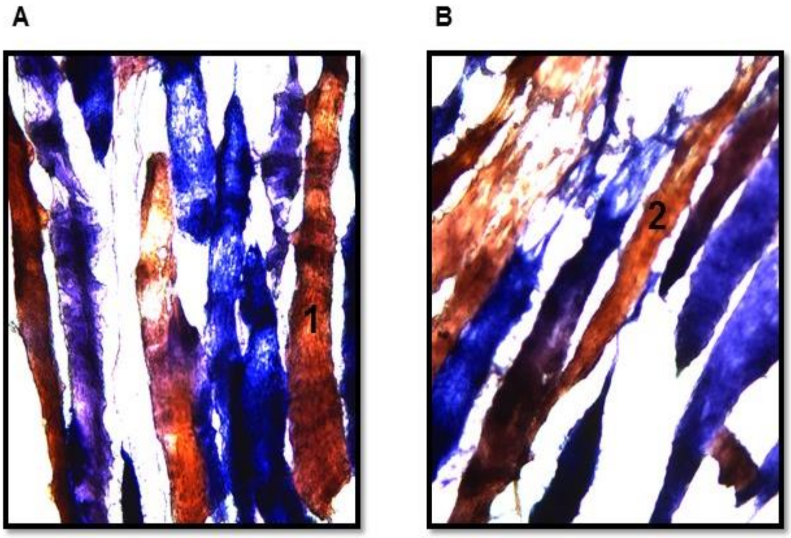

A-first longitudinal COX positive fiber marked with 1. B- second longitudinal COX positive fiber marked with 2.

Figure S3. Long Range PCR from single cells and longitudinal fibers showing full length gels

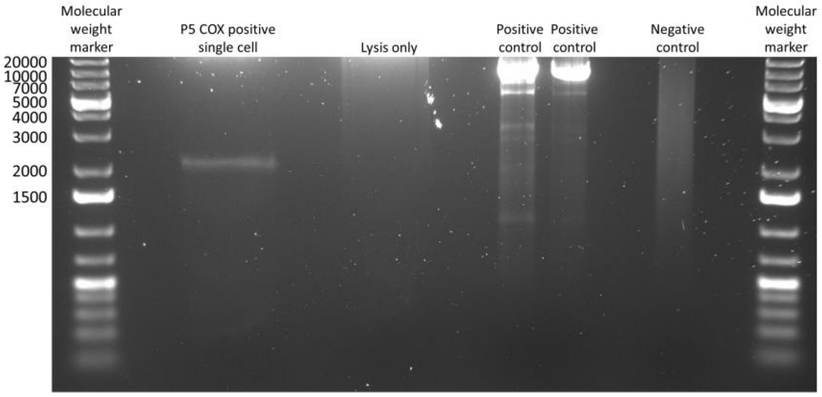

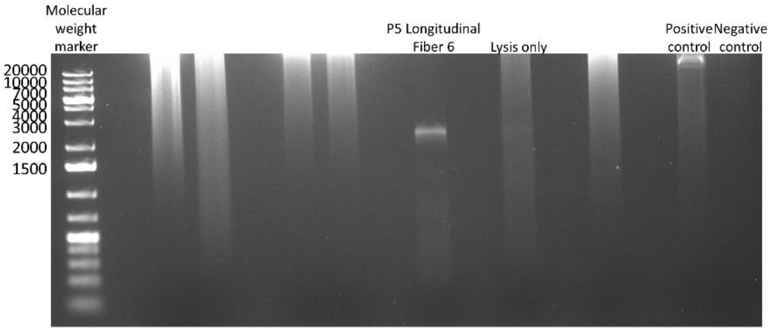

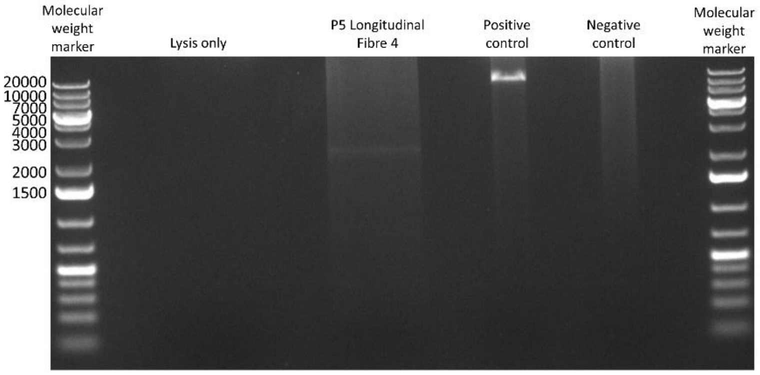

Long range PCR from patient 5 single cells and longitudinal fibers. The deleted molecule band is approximately 2500bp. The wild type molecule is approximately 10000bp.

Supplementary Table 1. Summary of the patients tested for long range PCR (LR-PCR), Southern blot (SB) and ddPCR.

Case	Age and Sex	Phenotype	LR-PCR in muscle DNA	SB in muscle DNA	ddPCR in blood DNA
P8	74y M	mitochondrial myopathy	weak multiple deletions	weak multiple deletions, 7kb-10kb	Negative
P9	47y F	CPEO	~3kb single deletion	single deletion 3 kb, 45%	Negative
10	26y M	Kearns-Sayre Syndrome	~7kb single deletion	single deletion 8kb, 30%	Negative
11	15y M	Kearns-Sayre Syndrome	single deletion	single deletion 6kb, 50%	Negative
12	21y M	Polyendocrinopathy	~7kb single deleion	single deletion 7kb, 30%	Negative
13	43y M	CPEO-plus	~7-8kb multiple deletions	multiple deletions 7-8kb, 50%	Negative
14	35y M	CPEO	~4kb single deletion	single deletion 4kb, 40%	Negative
15	54y M	CPEO	multiple deletions	multiple deletions 7-8kb	Negative
16	46y F	mitochondrial myopathy	multiple deletions	weak multiple deletions	5%
17	49y F	CPEO and exercise intolerance	~6.5kb single deletion	single deletion 6.5kb, 40%	Negative
18	36y M	Exercise intolerance, high CK, cardiac arrhythmia	multiple deletions	multiple deletions	Negative
19	52y F	Mitochondrial myopathy	multiple deletions	multiple deletions	Negative
20	59y F	Ptosis and exercise intolerance	~7kb single deletion	single deletion 7kb, 20%	3%
21	58y F	CPEO and mitochondrial myopathy	~2 kb single deletion	single deletion 2kb, 60%	Negative
22	<1y F	Pearson syndrome, lactic acidosis	blood ~5kb deletion	blood single deletion 5-6kb, 70-80%	Positive, 63.7%
23	16 F	Kearns Sayre Syndrome, T2basal ganglia lesions	Blood ~6kb deletion	blood, single deletion 6-7kb, 50-60%	Positive, 55.7%

F, female; M, male; y, years; LR-PCR, long range PCR; SB, Southern blot; ddPCR, Droplet Digital PCR. Results obtained on blood derived DNA are in red.

Supplementary Table 2. Primers and probes used in ddPCR experiments

ND1 Forward	5’CCCTAAAACCCGCCACATCT-3’
ND1 Reverse	5’-GAGCGATGGTGAGAGCTAAGGT-3’
ND1 Probe	5’-FAM/CCATCACCCTCTACATCACCGCCC/BHQ1-3’
ND4 Forward	5’-CCATTCTCCTCCTATCCCTCAAC-3’
ND4 Reverse	5’-CACAATCTGATGTTTTGGTTAAACTATATTT-3’
ND4 Probe	5’-HEX/CCGACATCATTACCGGGTTTTCCTCTTG/BHQ2-3’

Primer numbers refer to the mtDNA Genbank reference sequence NC 012920.1
